# Protocol for a randomized controlled trial investigating the effects of sleep extension on body weight and learning in children (More2Sleep)

**DOI:** 10.3389/fped.2026.1758117

**Published:** 2026-05-11

**Authors:** Cecilie H. Rasmussen, Nadia D. Neesgaard, Malene Skriver-Bayer, Caroline S. Muushardt, Eva Leedo-Townend, Jonas R. Bjørndal, Maria I. Rosenkrans, Andreas Wulff-Abramsson, Louise Mejer, Kathrine Skak Madsen, Line K. Johnsen, Hartwig R. Siebner, Nanette M. Debes, Chantelle N. Hart, Guy Plasqui, Anders Grøntved, Glen Nielsen, Poul J. Jennum, Jesper Lundbye-Jensen, Faidon Magkos

**Affiliations:** 1 Department of Nutrition, Exercise and Sports, University of Copenhagen, Copenhagen, Denmark; 2 Danish Research Centre for Magnetic Resonance, Department of Radiology and Nuclear Medicine, Copenhagen University Hospital—Amager and Hvidovre, Copenhagen, Denmark; 3 Department of Regional Health Research, University of Southern Denmark, Hvidovre, Denmark; 4 Department of Clinical Medicine, Faculty of Health and Medical Sciences, University of Copenhagen, Copenhagen, Denmark; 5 Department of Neurology, Copenhagen University Hospital Bispebjerg and Frederiksberg, Copenhagen, Denmark; 6 Department of Pediatrics, Copenhagen University Hospital—Herlev and Gentofte, Herlev, Denmark; 7 Center for Obesity Research and Education and Department of Social and Behavioral Sciences, Temple University, Philadelphia, PA, United States; 8 NUTRIM School of Nutrition and Translational Research in Metabolism, Department of Nutrition and Movement Sciences, Maastricht University, Maastricht, Netherlands; 9 Department of Sports Science and Clinical Biomechanics, University of Southern Denmark, Odense, Denmark; 10 Danish Center for Sleep Medicine, Department of Clinical Neurophysiology, Department of Health Technology, Technical University of Denmark, Rigshospitalet, Copenhagen, Denmark

**Keywords:** BMI, brain, cognitive function, learning ability, metabolism, obesity, sleep

## Abstract

**Background:**

Sleep is essential for children's health, cognitive development, academic performance, and overall well-being. Short sleep duration has been associated with several adverse outcomes, including an increased risk of obesity and impaired cognitive functions. Despite these associations, few studies have demonstrated causal links with these outcomes. Objective: To assess the effects of sleep extension on body weight regulation and learning ability in school-aged children with nocturnal sleep below recommendations for their age. Method: More2Sleep is a randomized, controlled, parallel trial involving 142 school-aged children (6–12 years old) who are “short-sleepers” (≤9 h/night). The children will be randomly assigned to a sleep extension group (by ∼45 min/night, achieved by going to bed 60–90 min earlier than usual; *n* = 71) or a control group (habitual sleep schedule; *n* = 71) guided by a family advisor for 12 weeks, followed by a 6-month inactive follow-up. All outcomes will be measured at baseline and 12 weeks. Selected outcomes will be reassessed at the 6-month follow-up to evaluate the sustainability of the intervention. Subgroups of children will undergo additional tests (substudy-I: metabolism and appetite; substudy-II: structural, functional, and diffusion MRI) to evaluate the mechanisms by which sleep may affect weight homeostasis and cognitive functions. Co-primary outcomes include the change in BMI z-score and learning ability from baseline to 12 weeks. Secondary outcomes include percent body fat, dietary energy intake, hunger and satiety, physical activity, energy expenditure, brain activity patterns, and cognitive and academic performance. Discussion: This study has broad implications as it can potentially inform future sleep recommendations and treatment strategies to reduce overweight/obesity and improve children's metabolic health and learning abilities.

**Clinical Trial Registration:**

https://clinicaltrials.gov/study/NCT06341179, identifier NCT06341179.

## Introduction

Sleep is essential to children's and adolescentś health and overall well-being (1–5). However, sleep duration among children and adolescents has declined over the past decades (6). Insufficient sleep is associated with a range of detrimental physical and mental outcomes, including increased risk of obesity (5, 7), inflammation (8), cardiovascular diseases (9–11), cognitive impairments (1), lower academic performance (1, 2, 12), and altered brain development (13).

Among these adverse outcomes, the relationship between insufficient sleep and obesity is particularly interesting. In parallel with a decrease in overnight sleep duration among children and adolescents (6), obesity rates have increased from 2% in 1990 to 8% in 2022 worldwide (14). In fact, there is a growing body of literature suggesting strong associations between short sleep duration and a greater risk of obesity in this age group (7, 15–19). Pooled results from cohort studies indicate that short sleep duration doubles the risk of overweight or obesity in children (7). Similarly, a meta-analysis found that children who sleep less than 10 h per night have nearly twice the odds of being obese compared to those who sleep longer (15). This association appears to be dose-dependent, with an additional meta-analysis demonstrating that each additional hour of sleep reduces the risk of overweight/obesity by 9% (16). Although these findings indicate that short sleep duration is a significant risk factor for weight gain and obesity early in life, the observational nature of these studies prevents inferences on causality.

Only a few experimental studies have investigated the effect of sleep duration manipulation on body weight homeostasis or eating behavior in school-aged children (20–22). A 3-week randomized crossover study in 37 children found that sleep restriction increased reported food intake (particularly sugary drinks), fasting leptin, and body weight relative to sleep extension. It also resulted in greater caloric intake in the evening (resulting in a delay in the timing of mean daily caloric intake and changes in sedentary and physical behaviors as well), albeit changes in physical activity were mixed (20, 23, 24). These results provide proof of concept that sleep duration is causally linked with obesity in children. They are, however, difficult to interpret as the two conditions (extension vs. restriction) differed by 141 min/night, which is unlikely to be achieved in real life. To further explore this, a subsequent parallel study of 78 children randomly assigned to either sleep extension or habitual sleep for 2 months (21). There were no significant changes in body weight or calorie intake between groups, but a combined analysis across groups showed that those who extended their sleep by at least 30 min/night consumed significantly fewer calories from fat and had a lower body mass index (BMI) z-score than those who did not increase their sleep (21). Secondary analyses further demonstrated that although there was not a direct effect of intervention on weight status, earlier bedtimes mediated the effect of intervention on child BMI z-score (25). Taken together, the findings of this study will support the idea that sleep extension may benefit weight management in children. However, additional experimental studies are needed to investigate the effects of sleep extension on body weight regulatory mechanisms in children.

Along with its impact on physical health, sleep duration is vital to brain and cognitive development, including learning ability, memory processes, and school performance (2, 12, 26–29). Short sleep duration has been consistently linked to poor school performance in children and adolescents (1, 2). Only few experimental studies have examined the role of sleep duration manipulation in cognitive development. In a crossover study, 77 children were randomized to 3 nights of 1 h sleep extension or restriction. Those who extended their sleep by at least 30 min improved their performance on digit forward memory and continuous performance tasks. In contrast, performance on a simple reaction time task significantly decreased in children who restricted their sleep by at least 30 min. This study also provided evidence that sleep quality (sleep onset latency, sleep percentage, night awakenings) is independent of sleep duration and may affect neurobehavioral functioning (30). Another study of 32 children with a very similar design (4 nights of 1-hour sleep extension or restriction after a week of habitual sleep in a crossover design) reported that children performed better on a battery of tests focused on emotion regulation, memory, attention, and math fluency after sleep extension vs. restriction (31). The results also demonstrated a noteworthy order effect: children who began with sleep extension performed better in memory and attention tasks than those who began with sleep restriction. Furthermore, exploratory findings by the authors showed that the overall difference in sleep duration between the two conditions was negatively associated with emotion regulation and aspects of attention related to inhibitory control.

Not only can sleep influence cognitive functions in children, but recent studies have also found that certain sleep behaviors (sleep timing, duration, and pattern) were related to gray matter volume in cortical and subcortical brain structures (32). A recent cohort study in preadolescents found that insufficient sleep (defined as less than 9 h/night) had adverse effects on both brain and behavior compared to those with sufficient sleep (33). Specifically, insufficient sleep had adverse effects on depression, thought problems, picture-vocabulary test performance, and crystalized intelligence. Furthermore, children with insufficient sleep also exhibited differences in cortical grey matter volume across 12 regions and in resting-state functional connectivity, especially in the basal ganglia, at baseline and at 2-year follow-up. A mediation analysis revealed that at baseline, the functional and structural brain measures mediated the effects of sleep duration on all behavioral measures. Longitudinally, the connectivity between the retrosplenial-temporal network and the right ventral dienchephalon mediated effects of sleep duration on picture-vocabulary test performance and crystallized intelligence (33).

The interconnectedness between sleep, weight status, and cognitive performance is illustrated by the results of a meta-analysis of 110 unique cohorts encompassing almost 600,000 healthy children and adolescents aged 5–17 years from 40 countries. This analysis indicates that shorter sleep duration is associated with higher adiposity indicators and poor academic achievement (5). However, as experimental studies are scarce, understanding the direction and magnitude of these associations and the underlying mechanisms remains elusive. Furthermore, it remains unclear whether extending sleep duration can mitigate the adverse effects of short sleep on weight status and cognitive function in children. This is crucial for developing effective strategies to improve children's health and overall well-being.

The More2Sleep study aims to fill this gap by investigating the potential beneficial effects of sleep extension by ∼45 min/night, achieved by going to bed 60–90 min earlier, on body weight homeostasis and learning ability in school-aged children with a shorter overnight sleep duration than recommended for their age (≤9 h/night). The secondary objectives are included to evaluate various relevant physiological processes that will provide mechanistic insights into the effects of sleep extension on body weight homeostasis, cognitive functioning, and academic performance.

## Methods and analysis

Methods reporting follows the *Standard Protocol Items: Recommendations for Interventional Trials* (SPIRIT) statement and the *Consolidated Standards of Reporting Trials* (CONSORT) guidelines. The complete SPIRIT checklist can be found in the online Supplementary Material.

### Study design

More2Sleep is a two-arm, parallel, randomized controlled trial in 142 children who are allocated in a 1:1 ratio to either an intervention group (*n* = 71, sleep extension) or a control group (*n* = 71, habitual sleep schedule). The study has one main level of participation (all 142 children) and two additional voluntary levels: substudy-I (*n* = 60; 30 children per group) and substudy-II (up to *n* = 142; 71 children per group).

**Figure 1:**
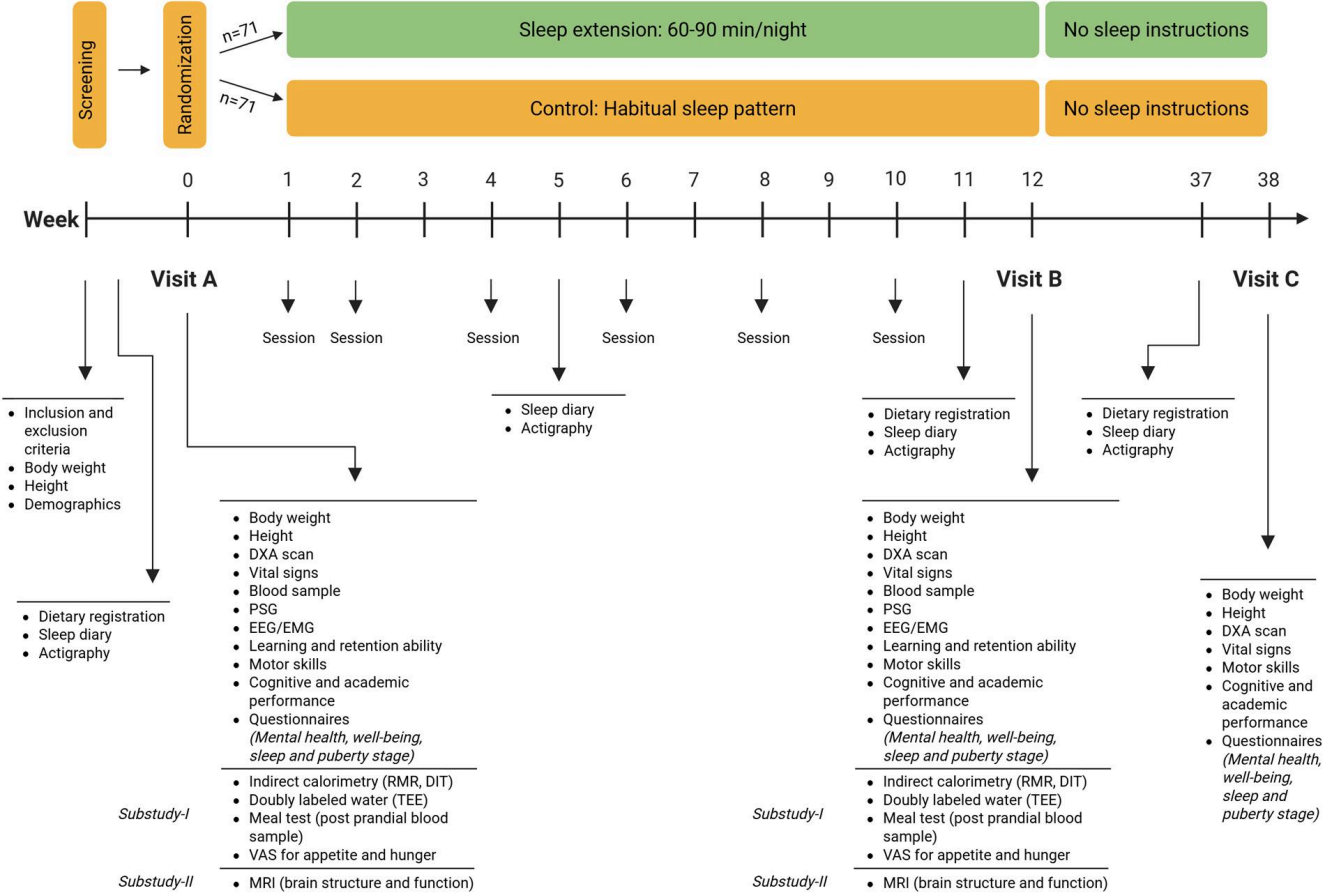
Overview of the study design. Children will be screened for eligibility during a screening visit. Randomization will take place after the completion of the baseline visit. There are three assessment visits: **(A)** (baseline), **(B)** (12 weeks), and **(C)** (6-month follow-up). In addition, at the beginning, middle, and end of the active intervention and at the end of follow-up, the children will wear an actigraph, and the parents will complete a sleep diary. Six sessions with a family advisor will be held during the first 12 weeks. Subgroups of participants will participate in substudy-I and/or substudy-II. DIT, diet-induced thermogenesis; DXA, dual-energy x-ray absorptiometry; EEG/EMG, electroencephalography/electromyography; MRI, magnetic resonance imaging; PSG, polysomnography; RMR, resting metabolic rate; TEE, total energy expenditure; VAS, visual analog scale. [Created in BioRender. Rasmussen, C. (2025) https://BioRender.com/a2d6dcr].

The study consists of a 12-week active intervention and a 6-month inactive follow-up (i.e., 38 weeks post-randomization). During this inactive follow-up period, no contact with the research team will take place. However, families in the sleep extension group will be instructed to continue putting their child to bed 60–90 min earlier. The overall study design is illustrated in Figure 1. All children will be assessed at three time points: baseline (Visit A), at the end of the active intervention (Visit B, week 12), and at the end of the 6-month follow-up (Visit C). During the intervention period, participants in both groups will attend 6 sessions with a family advisor, combining in-person and virtual formats over 12 weeks, to ensure consistent and equal contact frequency with the research team.

### Recruitment and screening

Participants will be recruited from the Capital Region of Denmark and Region Zealand through a multifaceted approach, including the Danish Civil Registration System (CPR registry), social media platforms, dedicated webpages, professional companies, flyer distribution in educational institutions and healthcare facilities, and word-of-mouth.

Following successful prescreening, the participating families will receive detailed written and oral information regarding the study. If they still wish to participate, the children's guardian(s) will sign an informed consent form and a data protection regulation form, including optional consent for future use of data and biological material. Verbal assent from the children will also be obtained. Thereafter, participants will be invited to a screening visit to evaluate the child's eligibility by obtaining anthropometric measurements, parent-reported sleep duration, medical history, and medication use. Provided that the child is eligible, a demographic questionnaire will be completed, and the family will be provided with a wrist-worn accelerometer for the child to wear and a paper-based sleep diary. They are instructed on how the child should wear the device and how to complete the sleep diary (parents on behalf of the child) for 7 days and 8 nights before Visit A. They are also given access to an online diet assessment tool and instructed on how to complete a 3-day food registration for the child during the same period on weekdays.

### Eligibility criteria

This study plans to recruit 142 children aged 6–12 years who are short-sleepers, that is, sleep 9 h/night or less (34, 35). Children will be excluded if they have any genetic, neurological, endocrine, or psychiatric condition that affects growth, metabolism, eating behaviors, cognitive function, or body weight; any sleep-related disorder; or are taking medication that could influence study outcomes. Additional exclusion criteria include an irregular school schedule, participation in other research studies, or neither parent being able to speak and understand Danish or English. If the child lives in two households, participation will be allowed only if both households can adhere to the intervention on school nights. For substudy-II, children will be excluded if they have metal implants and/or claustrophobia.

### Interventions

#### Sleep extension

Children and their parents will participate in a behavioral intervention, guiding the parent(s) to put their children to bed 60–90 min earlier at night while maintaining the same wake-up time in the morning (20, 21, 36). Previous studies have found that this adjustment is feasible and typically results in 40–45 min of additional sleep (21, 36). Parents of the participating children will be advised to alter only their children's bedtime and will not be provided with specific instructions regarding their children's dietary intake or physical and sedentary activity.

The behavioral intervention consists of 6 sessions delivered over a 12-week period. These sessions will be conducted in person or virtually, depending on the family's availability and to accommodate potential illness or other unforeseen circumstances. The first two sessions will take place within the first two weeks of the intervention. In the initial session, information about the child's sleep patterns is gathered, and a new sleep routine plan is developed in collaboration with the child and parent(s). Specifically, a cooperation agreement is established to support adherence to the new sleep routines. As part of this agreement, the family agrees to non-monetary, non-food-based rewards that emphasize quality family time, such as movie nights, board games, and outings that the child can look forward to. Rewards are scheduled to be given in one to two weeks, midway (week 6), and at the end of the intervention (week 12). Families may shift their child's bedtime by 60–90 min immediately or gradually increase the change over one week to reach the 60–90-minute goal. While both the parent(s) and child are involved in establishing the new routine, particular emphasis is placed on the child's wishes to foster a sense of ownership.

The family will receive guidance on sleep hygiene practices and effective behavioral strategies to enhance sleep time. These strategies include goal setting (e.g., bedtimes and wake-up times), self-monitoring using a sleep diary, problem-solving, preplanning, stimulus control (i.e., sleep hygiene recommendations), and positive reinforcement. Families will also be offered pictograms to support consistent bedtime routines, such as putting on pajamas, brushing teeth, reading a book, and turning off the lights. In addition, they will receive guidance on managing common bedtime challenges, such as the child repeatedly leaving his/her bed. They will also be instructed to avoid caffeinated drinks from 1:00 pm and refrain from using screens (e.g., mobile phones, tablets, and TVs) during the hour before bedtime. In the second session, the new sleep routines are evaluated with the family and adjusted, if needed.

The remaining four follow-up sessions will be conducted biweekly to reinforce the routines introduced in the initial sessions. One of these sessions will be held without the child being present, allowing the parent(s) to share their experiences more freely. Throughout these sessions, families will continue to be encouraged to use non-monetary, non-food-based rewards to motivate and foster compliance, with an emphasis on the quality time spent together, such as playing games or reading. The implementation of the new sleep routines will be evaluated at each session, and families will receive positive reinforcement or suggestions for adjustments if any challenges arise.

The final session includes a review of the collaboration agreement established in the initial sessions to evaluate the family's adherence to the agreed-upon routines and rewards. This session will also prepare the family for upcoming assessments and reinforce their continued commitment to follow sleep routines beyond the intervention period. Lastly, parents will also be asked to evaluate the tools and strategies used during the intervention period.

#### Control

The control group families will be instructed to maintain their children's habitual daily sleep routine, allowing diet and physical activity habits to change naturally. To equilibrate the potential influence of contact with the research team, the control group will have the same number and frequency of sessions (in-person and virtual). These sessions will be educational in nature and will focus on the general well-being of children and family relations (i.e., child independence, daily structure and routines in the family, and learning about emotional self-regulation), in addition to informing them about the different assessments at home (sleep diary, accelerometer, dietary registrations, and urine collection).

### Compliance

Compliance will be assessed using both a parent-reported sleep diary that records bedtime, sleep onset time, and wake-up time, and actigraphy data collected via a wrist-worn accelerometer, with the children wearing the accelerometer on the same days as the sleep diary is completed. Compliance is defined as an increase in sleep duration of ≥30 min/night after 12 weeks.

### Data collection and outcomes

The study involves various visits for participating families, including an information meeting, screening, assessment visits A and B (each spanning over 2 days), visit C, sessions with the family advisor, and additional visits for those participating in substudy-I and substudy-II. Data collection follows standard operating procedures and is detailed in Tables 1, 2. Most data are collected during the active intervention period. Participants who discontinue the intervention will be invited to attend an early termination visit. Participants who withdraw from the study during the active intervention will be invited to attend an early termination visit identical to Visit B, whereas those who withdraw during follow-up will be invited to attend a visit identical to Visit C.

**Table 1:** Overview of visits and assessments for the main study.

Method/assessment	Outcomes	At home	Visit A	At home	Visit B	At home	Visit C
		(Week −1)	(Week 0)	(Weeks 1–11)	(Week 12)	(Weeks 13–37)	(Week 38)
Method/assessment	Outcomes						
Anthropometry and metabolic health							
Anthropometric measurements	BMI z-score		X		X		X
DXA	Fat and lean mass and fat percentage, BMD, and BMC		X		X		X
Fasting blood sample	Clinical biochemistry: cardiometabolic risk factors, appetite-regulating hormones, sex hormones, and PBMCs.		X		X		
Puberty status	Tanner stage		X		X		X
Sleep							
Actigraphy	Sleep duration, movements, and awakenings	X		X		X	
Polysomnography	Sleep stages, duration, latency, awakenings, and arousals		X		X		
Stanford Sleepiness Scale	Alertness		X		X		X
Sleep Screen Questionnaire-Children and Adolescents (SSQ-CA)	Sleep duration, latency, awakenings, and tiredness/sleepiness		X		X		X
Learning and brain function							
Track-It and word memory test	Learning ability, including skill learning ability and explicit memory		X		X		
EEG/EMG	Muscle activity during skill learning, corticocortical functional connectivity, and corticomuscular connectivity		X		X		
School performance	Mathematics proficiency and Danish reading comprehension		X		X		X
Motor skills							
Pegboard test and handgrip dynamometer	Fine motor skills and muscular strength (gross motor skills)		X		X		X
Lifestyle behaviors							
3-day dietary record	Total energy intake, energy percentage of carbohydrate, fat, protein, and fiber, and meal timing	X		X		X	
Meal timing diary	Eating window duration	X		X		X	
Actigraphy	Total physical activity and time spent on MVPA	X		X		X	
Well-being and mental health							
KIDSCREEN-27	Total Health Related Quality of Life scores or subscale scores: physical well-being, psychological well-being, autonomy, parent relation, peers and social support, and school environment		X		X		X
Perceived Stress Scale for children (PSS-C)	Perceived child stress		X		X		X
Pediatric Quality of Life Inventory (PedsQL)	Total Health Related Quality of Life and Physical Health Summary score, Psychosocial Health summary score		X		X		X
Strength and Difficulties Questionnaire (SDQ)	Total difficulties score and subscale scores of emotional symptoms, conduct problems, hyperactivity/inattention, relationship problems, and prosocial behavior		X		X		X
Perceived Stress Scale (PSS)	Perceived parental stress		X		X		X

BMI, body mass index; BMC, bone mineral density; DXA, dual-energy x-ray Absorptiometry; EEG/EMG, electroencephalography/electromyography; MVPA, moderate-to-vigorous physical activity; PBMC, peripheral blood mononuclear cells.

**Table 2:** Overview of visits and assessments for substudy-I and substudy-II.

Method/assessment	Outcomes	Visits A and B	Visit B
		(Week 0)	(Week 12)
Method/assessment	Outcomes		
Substudy-I			
Fasting and postprandial blood samples	Clinical biochemistry: cardiometabolic risk factors and appetite-regulating hormones	X	X
Indirect calorimetry	Resting metabolic rate	X	X
	Diet-induced thermogenesis		
Doubly labeled water*	Total energy expenditure	X	X
Image-based appetite rating scale	Fasting and postprandial subjective level of hunger and satiety	X	X
Substudy-II (MRI)			
Structural MRI: MP2RAGE (T1) and T2-weighted imaging	Cortical thickness, volume, surface area, volumes of subcortical gray matter structures, myelination (R1)	X	X
Diffusion-weighted imaging	Brain gray and white matter microstructure	X	X
Resting-state fMRI	Brain functional connectivity	X	X

fMRI, functional magnetic resonance imaging; M2PRAGE: magnetization prepared 2 rapid acquisition gradient echoe.

* DLW urine samples are collected at home over a 14-day period following visits A and B.

#### Primary outcomes

##### BMI z-score (main study)

Fasting body weight and height are measured in duplicate to the nearest 0.1 kg and 0.1 cm, respectively, using a combined calibrated digital scale and stadiometer (Seca 285, Seca GmbH & Co. KG, Hamburg, Germany), with the children wearing underwear and a poncho (the weight of the poncho is known and subtracted). BMI (weight/height^2^) will be calculated, and normative age- and sex-specific reference data will be used to calculate the BMI z-score (37); using the WHO Anthroplus software for children aged 5–19 years.

##### Learning ability (main study)

The assessment of learning ability will be performed as motor skill learning and wordlist memory, involving implicit and explicit memory processes (38, 39). Motor skill learning is assessed using a computer-based sequential visuomotor task that requires both speed and accuracy. The children track visually displayed targets on a 24-inch computer screen placed in front of them by controlling the vertical position of a cursor using dynamic finger pinching movements (40s) (40–42). During the task, each target is displayed for 2s, and performance is calculated as a percentage of time on target score across all displayed targets. The task will involve the dominant hand determined by handedness preference.

After introduction to the task and a standardized familiarization procedure (1 min), the child will perform a baseline test (105 s), followed by four practice blocks of 2 min each, and then retention tests (immediate and 24 h later). Baseline and retention tests are identical. The test will assess overall 24 h learning, defined as the difference between the performance (percentage score out of 100) at baseline (day 1) and a delayed retention test 24 h later (day 2). This will be compared before and after the intervention. Performance will also be assessed immediately after the learning session to distinguish within-day from between-day (overnight) learning effects in secondary analyses (38, 39). Additionally, participants will perform a non-sequential variant of the motor task at delayed retention, enabling the distinction of sequence-specific and more general motor learning in secondary analyses. The procedure is identical at visits A and B. For both groups, we expect a repeated administration effect at visit B, but the learning task has not demonstrated ceiling effects in previous studies, meaning that repeated testing is feasible.

Explicit memory will be assessed using a word memory test with encoding in the main experiment and assessment with 2-min and 24-hour delayed recall. The encoding phase involves a 2-min period during which 20 words are displayed on a screen and read aloud in an automatized procedure. Explicit memory is assessed as the number of correctly recalled words (during 2 min) at delayed recall, i.e., total within and between session effect (range: from 0 to 20). All words are common nouns, and all words are replaced between visit A and visit B.

#### Secondary outcomes

##### Body fat percentage (main study)

Body fat percentage will be calculated from body composition data (fat mass, soft tissue lean mass, and bone mineral content) obtained by whole-body dual-energy x-ray absorptiometry (DXA; GE Lunar iDXA, GE Healthcare, Madison, Wisconsin, USA).

##### Dietary energy intake (main study)

Dietary energy intake and macronutrient consumption (fat, carbohydrate, and protein) will be estimated using averages of 3 days of diet recording, facilitated through an online dietary assessment tool, myfood24.org, which is available in Danish, English, German, French, Spanish, Norwegian, and Arabic, and has been validated for use with children (43). Children and their parents will be asked to record, in chronological order, all foods and drinks consumed over 3 days of the week. This will exclude Friday, Saturday, and Sunday (44) to align dietary intake with sleep duration during the school days. The parents will receive a MyFood24 link on each registration day. In MyFood24, parents can search for Danish and international food items in the Frida Fooddata database (v2 2017.20.02), the Livmedelsverket National Food Agency database, McCance and Widdowson v7, UK general brands, plus Tesco. The parents will be instructed to record dietary intake either as weighed amounts or using image-based portion sizes. For mixed dishes, parents can create recipes and record the portion consumed. Dietary records will be reviewed by study staff upon return and checked for unrealistic intakes or missing information, which will then be clarified with the parents. If no dietary registration has been completed prior to the visits, parents will be asked to complete a new registration within the following week. In addition, the estimated duration of the daily eating window will be assessed by instructing parents to record the times when their child starts and finishes eating on each registration day.

##### Physical activity and sleep (main study)

Actigraphy will be used to obtain objective measures of daytime physical activity, sleep-wake rhythmicity, and average sleep duration (45). Assessments will be conducted during four monitoring periods of seven days and eight nights each: in the week before visits A, B, and C, and at the midpoint of the active intervention. During each monitoring period, parents will complete daily sleep diaries recording the child's bedtime, estimated lights-off time, and wake-up time.

Actigraphy will be recorded using research-grade, triaxial devices (SOMNOwatch™ eco, SOMNOmedics AG, Germany) worn on the non-dominant wrist (46, 47). Devices will record at 32 Hz, and data will be summarized in 30 s epochs (48). Activity classification, including differentiation between active and sedentary behavior, will be based on movement acceleration, ambient light, and body temperature. Sleep-wake classification will be based on movement thresholds (48).

Lights-off and lights-on times will be recorded manually, either by event marker on the device or in the sleep diary. The sleep period will be defined as the interval between the first and last registered sleep epochs, measured from the registered lights-off to lights-on times. Sleep onset will be defined when movement/activity remains below a predefined threshold for 15 consecutive epochs (7.5 min), after which the full 15-epoch period will be backward-labeled as sleep (48). Wake onset will be defined when movement/activity exceeds the same threshold for four consecutive epochs, after which the full 4-epoch period will be backward-labelled as wake (48).

As the devices are not waterproof, they must be removed during bathing and swimming. Non-wear periods will therefore be expected and will be defined as ≥20 consecutive minutes with no activity detected on an*y* axis (49). Data from a seven-day monitoring period will be considered valid if the participant has at least 4 days of data with at least 16 h of wear time per day (49).

##### Brain activity patterns (main study)

Brain activity patterns will be measured at rest during learning and cognitive performance testing using full-cap, 64-channel electroencephalography (EEG; BioSemi, Netherlands) (50). EEG allows the recording of electrical brain activity through electrodes placed in a cap covering the head. Thus, it is possible to measure very small electrical variations associated with synchronization of neuronal activity. Additionally, electromyographic recordings (EMG; BioSemi, Netherlands) (50) will also be obtained from two hand muscles (m. first dorsal interosseus and m. abductor pollicis brevis) during the motor learning task. EEG measures, which serve as markers of neuroplasticity, will be analyzed for connectivity and overall brain activity, and these neutral measures will be related to behavioral measures of learning.

##### Cognitive and academic performance (main study)

Cognitive performance will be evaluated using standardized computer-based tests of attention, working memory, delayed memory, processing speed, and math and reading comprehension (51). Neuropsychological tests include a computer-based modified Eriksen Flanker Test to evaluate reaction time, response accuracy, and inhibitory control. Additionally, tablet-based working memory and attention tests will be performed using the Cambridge Neuropsychological Test Automated Battery (CANTAB; cambridgecognition.com). These include motor screening (MOT), spatial working memory (SWM), reaction time (RT), Multitasking (MTT), and sustained attention (RVP). Mathematics proficiency and reading comprehension will be evaluated using the Hogrefe Math Test (MGR) and the Hogrefe Danish Reading Comprehension Test, respectively.

##### Energy expenditure (substudy-I)

The resting metabolic rate (RMR) and diet-induced thermogenesis (DIT) will be measured using indirect calorimetry (Vyntus CPX, Intramedic A/S, Denmark). The children will come to the laboratory in the fasted state and breathe normally for 25 min under a transparent plastic hood to measure RMR (52). Subsequently, they will consume a standardized breakfast meal (*t* = 0). To estimate DIT, further assessments of energy expenditure will be conducted 45–70 min and 95–120 min after meal ingestion. The size of the test meal will be identical before and after the intervention for all children. If a child does not consume the entire meal at baseline, the leftovers will be weighed, and the meal will be adjusted accordingly after the intervention. DIT will be calculated as the incremental area under the curve of energy expenditure over time and will be expressed as absolute calories (kcal) and percentage of ingested calories (%) (53). Blood samples (10 mL) will be collected in the fasting state and 2 h after the completion of the meal test.

Total energy expenditure (TEE) will be assessed using doubly labeled water (DLW), that is, water enriched with two stable isotopes of oxygen and hydrogen (^18^O and ^2^H) (54–56). On the first day of visits A and B, the children will provide a baseline urine sample and drink 50 mL of DLW. The first post-DLW urine sample will be collected the following morning, and 6 additional urine samples will be collected at home over the next two weeks. The DLW method is based on the differential rates of 2H and 18O excretion to estimate the rate of carbon dioxide (CO_2_) production (57). CO_2_ is the end product of human oxidative metabolism and is thus a direct metric of energy expenditure under free-living conditions (58).

##### Subjective feelings of hunger and satiety (substudy-I)

Subjective feelings of hunger and satiety will be obtained using an image-adapted 1–5 point rating scale, called “Teddy the bear”, which has been validated for school-aged children (59). The rating scale will be completed in the fasting state, and again at 1 and 2 h after consuming a standardized breakfast meal.

#### Exploratory outcomes

##### Sleep quality, architecture, and patterns (main study)

Sleep quality and architecture will be evaluated using polysomnography (PSG; HomeSleepTest REM+, SOMNOmedics AG, Germany). In addition to actigraphy, PSG objectively assesses sleep stages, total sleep time, latency, awakenings, and arousal. PSG testing will be conducted at home between days 1 and 2 at visits A and B. PSG data will be recorded using headband equipment, which the parents will mount at home in the evening on the first day and remove the next morning on the second day. Self-reported data on sleep duration, latency, awakenings, and daytime tiredness/sleepiness will be collected using the Scandinavian Sleep Questionnaire-Children and Adolescents (SSQ-CA) (60).

##### Mental health and overall well-being (main study)

Mental health and well-being will be assessed using several questionnaires. Measures of mood, fatigue, subjective sleepiness, and circadian rhythm, which are domains potentially influenced by sleep extension, will be assessed using the Stanford Sleepiness Scale (61) and the SSQ-CA (60).

Overall well-being and health-related quality of life will be evaluated using the parent-reported Pediatric Quality of Life Inventory (PEDSQL) (62) and the child-reported KIDSCREEN-27 questionnaire (63–65).

Mental health will be assessed using the parent-reported Strengths and Difficulties Questionnaire (66). Stress levels are measured using the Perceived Stress Scale for Children (67) and the Parental Stress Scale for parents (68).

##### Motor skills (main study)

Fine motor control will be assessed using the Purdue pegboard test (Model 32020, Lafayette Instrument Company, USA), and muscular strength will be evaluated using a handgrip strength test (BIMS Digital Grip Dynamometer, Baseline Evaluation Instruments, USA).

##### Blood biomarkers (main and substudy-I)

In the main study, blood samples (∼10 mL) will be collected in the fasting state by venipuncture to measure parameters of glucose homeostasis (e.g., glucose and insulin), lipid profile (e.g., triglyceride, low- and high-density lipoprotein cholesterol), inflammation markers (e.g., C-reactive protein), appetite-regulating hormones (e.g., leptin and ghrelin), sex hormones (e.g., estrogen and testosterone), and isolated peripheral blood mononuclear cells. For children participating in substudy-I, an additional 2-hour blood sample (∼10 mL) will be collected after the meal test.

##### Brain structure, activity, and connectivity (substudy-II)

Participants will undergo whole-brain structural, functional, and diffusion MRI on a 3 Tesla Siemens Magnetom Vida scanner (Siemens, Erlangen, Germany) using a 64-channel head coil at the DRCMR. Prior to scanning, children will be familiarized with the MRI environment using an MRI simulator. High-resolution T1-weighted and T2-weighted images will be acquired using 3D magnetization prepared 2 rapid acquisition gradient echo (MP2RAGE) (69) and 3D T2 SPACE sequences. These structural images will be used to quantify apparent cortical thickness, cortical volume, surface area, subcortical volumes, and structural covariance. In addition, the MP2RAGE provides an R1 map, which is sensitive to myelin content, and may therefore be relevant for assessing plastic changes related to the sleep extension intervention. Multi-shell diffusion MRI will be used to map the microstructural characteristics of the white matter and subcortical grey matter regions and to assess anatomical connectivity. Functional MRI will be collected during passive movie watching, allowing investigation of functional connectivity in a naturalistic setting (70).

#### Other measures

##### Socioeconomic status (main study)

Information on income, parental education, employment status, ethnicity, and immigration background will be obtained through a demographic questionnaire completed by parents during screening. These parameters will be used to classify children and their families into high or low socioeconomic strata (binary) to examine their potential influence on study outcomes and to tailor the intervention within each family's framework.

##### Puberty stage (main study)

A short questionnaire completed by the parents will assess the child's puberty stage (I-5) according to the Tanner stage classification method (71, 72), based on genital development and pubic hair status in boys, and breast development and pubic hair status in girls.

### Sample size determination

Sample size calculations were designed to achieve 80% statistical power (corresponding to a type II error of 0.2). Two-sided hypothesis testing with a type I error rate of 2.5% (i.e., *P* ≤ 0.025) was chosen because there are two co-primary outcomes, and a 20% dropout rate over 12 weeks was assumed.

A previous study on sleep extension in children aimed for moderate-to-large effect sizes, Cohen's *d* = 0.58, based on preliminary data (21). This study aims to achieve a similar effect size, resulting in an absolute difference in the final BMI z-score of 0.52 units between groups. We consider this effect size to be appropriate and clinically relevant, as studies of lifestyle interventions in children have demonstrated important clinical benefits with decreases in BMI z-score of 0.25–0.50 units (73).

In a previous study of sleep extension in children, including children of any weight status, a mean BMI z-score of 0.9 was reported, with a standard deviation of 0.9 (21). Based on these data, we assume a mean baseline BMI z-score of +1.0. Under these assumptions, we estimate we will need 114 children assigned in a 1:1 ratio between the active and control groups to detect an effect size of d = 0.58. Although sleep intervention studies in children report dropout rates below 4% (21), we based our calculations on a more conservative 20% attrition rate (i.e., five times higher than previously observed but more realistic for our longer study duration and more demanding assessment). We estimated that we would need to enroll 142 children (for 114 completers).

Considering learning and memory, we defined our co-primary outcome as overall 24 h learning. Previous studies on sleep extension in children have found moderate effect sizes for improved performance in the cognitive domain, corresponding to similar effect sizes for the academic performance domain (31). We have not found studies investigating the effects of sleep or sleep extension on 24 h learning effects. In recent experiments, we found that overnight sleep is related to total 24 h skill learning in children and that children benefit from offline learning effects compared to adolescents and young adults (41). Based on the mean performance and standard deviation at baseline among school-aged children aged 8–10 years, we anticipate that a sample size of 142 will allow us to detect a moderate-to-large effect size in the learning and memory domain at *P* ≤ 0.025, with 80% power.

The two substudies are exploratory studies designed to provide mechanistic insights. Therefore, we did not conduct power calculations separately for the two substudies. Instead, we selected the respective sample sizes based on previous studies using DLW to measure changes in TEE, which typically include groups of 20–60 children (74–76), and longitudinal studies of MRI-determined changes in brain structure and connectivity, which typically recruit 47–78 children per group (77–80) and rarely more (33).

### Randomization and blinding

After completion of baseline assessments (Visit A), the children will be informed of their group allocation by receiving a sealed envelope. The children will be randomized in a 1:1 ratio to the sleep extension or control group. To ensure balance between the two groups, randomization is stratified by sex (male or female), age (≤9 or >9 years), and participation level (main study only; main+substudy-I; main + substudy-II; main + substudy-I and -II), with each combination defining a stratum. The allocation is determined by a computer-based algorithm implemented in an Excel file. The algorithm is configured such that the probability of allocation depends on the current distribution of participants across groups, thereby maintaining balance between the sleep and control groups and within each stratum. This file is stored as a restricted-access file available only to designated staff for the randomization task. The allocation is subsequently recorded in an electronic case report form within the Research Electronic Data Capture (REDCap) system.

The behavioral nature of the intervention precludes blinding of children and their parents. Nonetheless, staff assessing outcomes and analyzing the data will be blinded to group allocation.

### Data management

Data will be recorded in electronic case report forms (CRF) in a secure REDCap database or on paper CRF. Data recorded on paper CRFs will be converted to electronic records within 5 days. Access to the REDCap database is restricted to authorized staff. Participants are assigned a unique study identification number to ensure de-identification of research data. To ensure data control, REDCap's built-in setup is used, which automatically notifies study staff about missing data. Source data from DXA scans, ventilated hoods, MRI, PSG, and analysis of biological material are registered in the relevant device interface software. All personal data and biological material are handled confidentially and stored in accordance with applicable law, GDPR, and the Danish Data Protection Act. (No. 502 of 23rd May 2018). For data collected from external sources (e.g., MyFood24 and CambridgeCognition), data are handled in accordance with data processing agreements. Study-related monitoring and audits by the principal investigator, the Institutional Review Board/Independent Ethics Committee, or regulatory inspections will be permitted. Direct access to the source data, CRFs, and other relevant materials will be provided. Source data will be cleaned and analyzed independently for each parameter by staff members blinded to group allocation. After the extraction of predefined parameters, the datasets will be merged into an analysis dataset using a secure data management platform.

### Statistical analysis

#### Baseline characteristics

Results for baseline characteristics will be reported as mean (standard deviation) for normally distributed variables and as median (interquartile range) for non-normally distributed variables. Categorical variables will be reported as frequency counts and percentages.

#### Primary analysis

The effect of the intervention will be evaluated using linear mixed-effects models (LMMs) estimated with restricted maximum likelihood (REML), allowing inclusion of all available observations in the statistical model without imputations.

The LMMs will be performed with fixed effects for group, time, and the group-by-time interaction, with participant included as a random effect. The primary model will include baseline and 12-week measurements. An extended model with 3 time points (baseline, 12 weeks, and 6 months) will be developed to assess the sustainability of effects over the follow-up period. These models will be adjusted for stratification variables and baseline pubertal stage, included as fixed main effects. Model assumptions will be evaluated using residual diagnostics. If model assumptions are not met, appropriate transformations will be considered.

The primary analysis will be conducted using an intention-to-treat approach, including all randomized children according to their group allocation, regardless of whether or not they complete the study.

#### Sensitivity analyses

Sensitivity analyses will be restricted to completers only and include a per-protocol analysis. This analysis will include only children from the sleep extension group who complete the study and extend their sleep by at least 30 min per night after 12 weeks. This amount of sleep extension is clinically relevant for outcomes related to body weight regulation and cognitive function (21, 30).

#### Exploratory analyses

In an exploratory analysis, interaction terms between group allocation and sex and between group allocation and BMI status will be included in the linear mixed models. Additionally, correlation and regression analyses will be used to evaluate the continuous association between changes in sleep duration and the outcomes.

Two-sided *P*-values ≤ 0.025 will be considered statistically significant for the primary outcomes. For secondary and exploratory outcomes, *P*-values ≤ 0.05 will be considered statistically significant. Statistical analyses will be conducted using IBM SPSS Statistics and R.

## Discussion

One of the main strengths of the More2Sleep study is its multidisciplinary approach, as it assesses two critical domains of children's health: body weight regulation and learning ability. The primary outcomes are complemented by several secondary outcomes expected to provide valuable insights into the mechanisms underlying the effects of sleep extension (e.g., energy expenditure, metabolic risk factors, cognitive performance, and brain function and structure). The findings of the study will provide a better understanding of the potential causal links between short sleep duration, body weight homeostasis, metabolism, and learning ability in children. Another strength is the inclusion of a large sample of school-aged children who sleep less than recommended, which increases our ability to detect a clinically relevant change and enhances the applicability of our findings.

We have designed the intervention (sleep extension and control) to include the same number and frequency of sessions with the family advisor in both groups. The content focusing on providing the whole family with guidance, motivation, and support will help achieve the required sleep extension. Previous studies have shown that collaboration between children and parents is important for adherence to similar behavioral interventions (81, 82). The frequency of meetings will also allow us to monitor adherence, provide support, and add or adjust strategies at home as needed. Combining in-person and virtual sessions can ease the logistical burden on families while still allowing progress to be tracked.

Additionally, the study design includes a dual-compliance assessment of sleep using parental reports and child actigraphy. This is important, as a previous study found that both parents and children tend to overestimate sleep duration, especially the time spent awake at night, compared with objective measures (83). Because parental reporting may be influenced by awareness of the intervention, reported adherence may be biased. However, this potential bias can be assessed using objective actigraphy analyses.

The behavioral nature of the intervention may introduce additional bias. Since the behavioral nature of the intervention precludes blinding of families and the family advisor, this may introduce performance bias if behaviors other than sleep are influenced during the study. To minimize the impact of this bias on our outcomes, both groups will follow a similar schedule of visits and interaction with study staff, and the same family advisor will be involved across groups to ensure consistency in contact and guidance. Furthermore, study staff who will assess outcomes and perform analyses will be blinded to group allocation, and several outcomes will be objectively measured, reducing the risk of detection bias. Nevertheless, it cannot be ruled out that general well-being content in the control group may influence behaviors such as diet, physical activity, or learning.

Another limitation is that even though More2Sleep has a longer duration than previous studies investigating the effect of sleep manipulation on children's weight (20, 21, 23, 24), cognitive functions, and academic performance (30, 31), the timeframe may still be inadequate to detect significant changes in body weight and learning abilities, which are outcomes typically affected on very long timescales.

Another consideration concerns which activities additional sleep may replace. Results from two systematic reviews, mainly focusing on sleep quantity, indicate a negative association between sleep duration and screen time in children and adolescents (84, 85). This highlights that the lack of assessment of screen use in this study may be a potential limitation.

Additionally, several questions remain about the role of sleep in children's health, which are beyond the scope of this study. One is defining the minimum amount of sleep required for optimal health, which in itself can be defined in various ways, for example, in relation to physical or mental performance (86). While guidelines on recommended sleep typically refer to the entire pediatric population within a certain age range, individual sleep needs likely vary widely and can be influenced by inter- and intra-individual factors (86). The importance of sleep quality vs. sleep duration also requires further clarification (30), but measuring sleep quality is more complicated. Although sleep quality will be evaluated in this study using PSG, these measurements will capture only one night's data and may not accurately reflect the child's typical sleep patterns.

Another limitation of this study is the lack of temporal information on which outcome (i.e., body weight or learning) improves first. While we will explore the underlying mechanisms of the potential effects of sleep on body weight homeostasis and learning ability, we will not be able to disentangle the interrelatedness between overweight/obesity and suboptimal cognitive functioning. We expect sleep extension to be accompanied by positive changes in both domains; therefore, we cannot conclude whether improvements in body weight regulation, in themselves (i.e., independent of sleep), may lead to improved cognitive functions, and vice versa. This highlights the need for further research to clarify causal pathways.

## Ethics and dissemination

The study (reference number: H-23063352) has been approved by the Danish Research Ethics Committee on 02 February 2024. Additional protocol amendments are submitted to the committee and updated in ClinicalTrials.gov on an ongoing basis. The study will be conducted in accordance with the ethical principles outlined in the current version of the Declaration of Helsinki (Fortaleza, Brazil, October 2013), the principles of the International Conference on Harmonization E6 Good Clinical Practice (ICH-GCP) R2, and all applicable local regulatory requirements.

All children participating in the main study (visits A, B, and C) will receive a gift certificate per completed visit. All children will receive a commercial activity smartwatch at the end of the 12-week active intervention. Children participating in substudies will receive an additional flexible gift certificate.

While the study intervention poses no direct risks, participation in clinical research may disrupt everyday life, especially for families with young children, thus requiring time for study procedures. Special attention will be paid to the children's responses and reactions throughout the study, and participation will be terminated if a child shows unwillingness to continue.

Certain procedures may carry minor risks. There is a minor risk of slight pain and bruising for blood samples, although local anesthetic patches will be used to minimize discomfort. A maximum of 20 mL of blood is collected from each of the participating children, and 40 mL from children participating in Study I. The DXA scan involves minimal radiation exposure (0.011 mSv), with only one rescan permitted per visit if the initial scan fails.

The DLW method is safe for use in humans of both sexes and all ages (56). The weighed dose of DLW contains a measured enrichment of about 6.6 atomic % ^2^H and 10 atomic % ^18^O. This results in an initial excess body water enrichment of 100–120 ppm for ^2^H, which is <0.2% of the threshold above which side effects and toxicity may manifest. No physiological effects have been detected even at body water enrichments of ∼60% ^18^O (56).

Wearing the actigraphy device, polysomnography equipment, and surface electrodes used in EEG and EMG may cause skin irritation. Additionally, in rare cases, EEG-compatible gel may cause an allergic reaction in particularly sensitive individuals. EEG and EMG are safe for use in children. If skin irritation is observed, electrodes and gel are removed, and these measurements are omitted.

MRI is a non-invasive imaging method with no known biological hazards and has been approved for use in children. To minimize distress and motion, children will be acclimatized to the MRI environment using a scanner simulator. Scanning will be terminated immediately if a child shows signs of discomfort or requests to stop.

Finally, to ensure participant safety, vital signs, including resting blood pressure, heart rate, and body temperature, will be assessed at all main visits (A, B, and C). Potential adverse events will be assessed during the study period. If the child's general condition precludes continuing the study, as determined by the study personnel or the medical expert, participation will be discontinued. Participation will also be discontinued in cases of significant non-compliance, lack of cooperation, or unforeseen events that make the child ineligible to participate. Additionally, all participants are insured in accordance with national legal requirements in the event of injury resulting from study procedures.

Regardless of the outcome, the results of the study will be published in peer-reviewed journals and presented at scientific conferences.

## Conclusion

More2Sleep is designed to determine the effect of a pragmatic sleep extension intervention on body weight regulation and learning ability in children who sleep less than the recommended for their age and sex.

This study will contribute evidence about the effects of sleep extension on children's health and well-being. The impact of this research is potentially considerable and multi-dimensional. In terms of research, this study may inspire future studies on sleep manipulation in childhood obesity, neurophysiology, and cognitive function. In terms of clinical practice, it may contribute to the development of sleep-based treatment strategies or inform future sleep recommendations for school-aged children. Perhaps more importantly, in terms of societal impact, this study has the potential to provide an easy way for parents to improve their children's body weight and learning abilities, which will accompany them for the rest of their lives.
